# A Novel Framework for the Design of Minimized Epigenetic Clocks Using the Analysis of DNA Methylation Heterogeneity

**DOI:** 10.3390/ijms26115051

**Published:** 2025-05-23

**Authors:** Stanislav E. Romanov, Dmitry I. Karetnikov, Darya A. Kalashnikova, Denis E. Polivcev, Yakov A. Osipov, Daniil A. Maksimov, Polina A. Antoshina, Viktor V. Shloma, Ekaterina M. Samoilova, Alina A. Ivanova, Rustam F. Karimov, Artem N. Tkalin, Alexander A. Shevchenko, Vladimir A. Kalsin, Vladimir P. Baklaushev, Petr P. Laktionov

**Affiliations:** 1 Epigenetics Laboratory, Department of Natural Sciences, Novosibirsk State University, 630090 Novosibirsk, Russia; romanov@mcb.nsc.ru (S.E.R.);; 2 Institute of Molecular and Cellular Biology, Siberian Branch of the Russian Academy of Sciences, 630090 Novosibirsk, Russia; polonium@mcb.nsc.ru (P.A.A.); shloma@mcb.nsc.ru (V.V.S.); 3 Federal Research Center Institute of Cytology and Genetics SB RAS, 630090 Novosibirsk, Russia; 4 Engelhardt Institute of Molecular Biology, Russian Academy of Sciences, 119991 Moscow, Russia; 5 Federal Center for Brain and Neurotechnologies, Federal Medical and Biological Agency of Russia, 117513 Moscow, Russia; 6 Federal Scientific and Clinical Center for Specialized Types of Medical Care and Medical Technologies, Federal Medical and Biological Agency of Russia, 115682 Moscow, Russia; 7 Department of Medical Nanobiotechnology, Medical and Biological Faculty, Pirogov Russian National Research Medical University, Ministry of Health of the Russian Federation, 117997 Moscow, Russia

**Keywords:** DNA methylation heterogeneity, epigenetic age, bisulfite sequencing, eAge clocks, mesenchymal stem cells

## Abstract

Despite the significant progress made in the development of epigenetic age (eAge) clocks designed to estimate the various aspects of aging, currently available models, generated using large DNA methylation microarray datasets, still cannot fully address the issues of batch effects and technical variation. This hinders the use of the publicly available eAge clocks in routine laboratory practice, and it motivates the development of cost-effective, custom epigenetic clocks that are tailored to the given biological subjects and research methods. In this study, we analyzed the local DNA methylation of mesenchymal stem cell samples during culture expansion using high-throughput targeted bisulfite sequencing (BS-seq). Using the obtained data, we trained a minimized eAge model based on a Random Forest Regression with Leave-One-Out Cross-Validation, which determines cell passage with good performance (MAE 1.094 and R^2^ 0.897) and which is comparable to previous solutions. Using the advantage of BS-seq to analyze consecutive CpGs methylation patterns, we demonstrated that combining the analysis of average DNA methylation levels with local methylation heterogeneity scores—thereby reflecting stochastic DNA methylation dynamics—can improve the quality of the epigenetic clock models. Therefore, we propose a research strategy for creating customized epigenetic clocks using targeted BS-seq and provide a mechanistic conceptualization of how information on longitudinal changes in DNA methylation patterns can potentially be used for the assessment of specific aging aspects.

## 1. Introduction

Allogeneic and autologous human mesenchymal stem cells (MSCs) are a promising component in regenerative and immunomodulatory cell therapy [1]. However, their practical application in the reported clinical trials entails extensive in vitro expansion to produce the hundreds of millions of cells that are required per procedure [2]. The long-term in vitro expansion of primary non-immortalized cells is inevitably linked to replicative senescence, which is caused by critical telomere shortening and accompanied by the alteration of morphology, an impaired metabolism, the induction of pro-inflammatory secretory phenotype, and a gradual decrease in proliferative potential, which eventually leads to irreversible cell cycle arrest [3,4]. Therefore, the duration of cell culture, alongside the age of the donor, might have a significant impact on the yield and quality of the cell preparations, requiring the control of that aspect [4,5]. Due to the intrinsic asynchronous and heterogeneous nature of cellular senescence, there is a current lack of a unique and universal marker to track it [6,7]. There are several described senescence-associated markers, including but not limited to the activation of the cell cycle inhibitors p16^ink4a^ and p21^Cip1^; the induction of senescent-associated β-galactosidase (SA-β-gal); telomere attrition; the downregulation of *LMNB1*, which affects nuclear integrity; decline of the Ki-67 marker of proliferation; and the emergence of DNA damage markers and pro-inflammatory components of senescence-associated secretory phenotype (SASP) [6,7,8]. However, none of these markers are exclusive to senescence, thereby necessitating their combined use in experimental studies [6]. In addition, most of them are not sensitive enough to capture early senescence dynamics and, therefore, they are only detectable at the later stages [9,10]. There are alternative approaches to track senescence leverage integrative data analysis by means of machine learning algorithms, including high-throughput morphological cell profiling, deciphering specific transcriptomic signatures, or DNA methylation patterns, with the latter being the currently most widely used [11,12,13,14].

DNA methylation is one of the key epigenetic marks that largely determine gene expression, genome stability, and the structure and spatial organization of chromatin [15]. Cell-type specific DNA methylation patterns might serve as proxy metrics of the current functional cellular state, reflecting normal development or pathological processes [16,17]. Both in vivo and in vitro aging are accompanied by characteristic changes in DNA methylation patterns, and their assessment forms the basis of epigenetic clock (eAge) algorithms, which allow for predicting age, lifespan, and age-related health risks [18,19,20]. Technically, such analysis involves the profiling of 5^m^C DNA methylation across dozens to thousands of CpG sites [14]. The first eAge clocks were designed for humans, followed by other mammalian species and vertebrates [14,21,22,23]. The most popular human eAge clocks analyze several hundred CpGs using microarray technology [18,20,24]. More affordable minimized eAge algorithms assess DNA methylation at individual CpGs by bisulfite conversion followed by real-time PCR or its variations, pyrosequencing, or targeted NGS sequencing, but to some extent, they sacrifice the ability to detect the impact of various factors influencing aging [14,25,26,27].

It is noteworthy that widely used microarrays or pyrosequencing generate the average DNA methylation values of individual CpG, thereby missing the information on the consecutive CpGs methylation pattern within individual DNA molecules. At the same time, the concordance of the methylation state between adjacent CpGs determines the concept of DNA methylation heterogeneity, as well as the diversity of the DNA methylation patterns that can be expressed with a single number within the sample heterogeneity (WSH) score [28]. Depending on its definition, various WSH metrics can be used to measure cell-type diversity or the randomness of patterns, stochastic erosion, or the stability of methylation, as well as to identify allele-specific methylation [28,29]. In some cases, the analysis of DNA methylation heterogeneity, rather than differentially methylated CpGs, allows for the identification of functionally significant genomic regions, e.g., to develop cancer diagnostics [30]. Recently, we proposed an approach for developing eAge models based on WSH metrics and suggested that its use can improve the performance of minimized epigenetic clocks [31].

One of the major advantages of publicly available pre-trained eAge clocks is the large volume of training datasets that should enable models to make more reliable predictions [18,32,33]. Unfortunately, despite this, they might generate distorted estimates when researchers encounter various sorts of batch effects and technical variation [34,35]. While there is no doubt that epigenetic clocks are a useful tool, their application in routine experiments is also complicated by the challenge of transitioning from microarray technology to faster and cheaper alternatives, e.g., used in minimized eAge clocks. For example, according to published data and our own experience, eAge algorithms trained on microarray datasets are prone to large prediction errors in cases where the test samples are analyzed with next-generation sequencing [36]. Although the method switching problem can be solved by linear adjustments [36], another limitation might arise when a specific eAge algorithm that is not sufficiently studied in terms of DNA methylation, and for which large DNA methylation datasets are not available, is required for the biological object (cell type, organism, etc.). In this regard, a viable solution might be the creation of a custom eAge clock that (1) is tailored to biological (e.g., population, cell type-specific, etc.) and technical (method of analysis, sample preparations, etc.) variations in the exact experimental setup and (2) might be built on the limited training datasets that might be generated in a single study.

Here, we used the in vitro replicative senescence of MSCs as a model to build a framework that can facilitate the development of a minimal epigenetic clock using targeted bisulfite sequencing (BS-seq). We show that the high-throughput analysis of four genomic regions can be sufficient to construct an adequate model to track the duration of in vitro cell culture with a low scatter of the predicted and actual passage. Moreover, we tested the conceptual applicability of the framework, which uses the analysis of both the DNA methylation level and heterogeneity to design the minimized eAge clocks. We found that including the average methylation level and WSH metrics as independent covariates can improve the performance of regression models to predict time in culture and chronological age.

## 2. Results

### 2.1. Simulation of the Design of Minimized Microarray-Based Cultural Age Clocks

**Figure 1:**
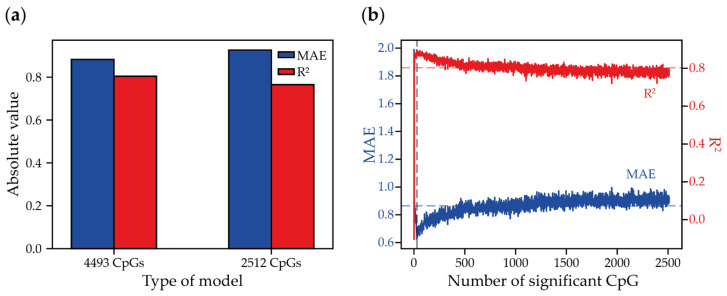
Predictive model efficiency depending on the number of CpG sites used. (**a**) MAE (blue) and R^2^ (red) for two models: 4493 passage-correlated CpGs and 2512 significant CpGs. (**b**) The change in R^2^ and MAE depending on the number of selected significant CpG sites. The gray vertical dashed line indicates the number of CpG sites at which the best MAE and R^2^ values were achieved (*N* = 28). The blue and red dashed horizontal lines indicate the average MAE and R^2^ values for all the models (0.865 and 0.803, respectively).

To set the comparative benchmark for the minimized eAge clocks, predicting the passage of MSCs, we first performed the in silico design of the DNA methylation microarray-based model. An extensive search for the published MSC DNA methylation microarray data, supported by the information on the cell passage and donor’s age (when applicable), allowed us to collect a dataset comprising 48 samples (Table S1) [37,38]. Only the datasets generated using Illumina Infinium HumanMethylation450 BeadChip or newer versions of the platform were considered, as the former platform only covers around 0.1% of human genome CpGs [39]. Next, we selected 4493 CpGs whose DNA methylation level correlated with the cell passage numbers (Pearson’s correlation, *p*-value-adj < 0.05). To build the model and avoid overfitting, we applied Random Forest Regression (RFR) with Leave-One-Out Cross-Validation (LOOCV). The model was built on the beta-values of 4493 CpGs and predicted passage numbers with a mean absolute error (MAE) of 0.882 and R^2^ equal to 0.804 (Figure 1a). To minimize the model, we iteratively built RFR models with LOOCV to filter out the variables with null significance, resulting in the selection of 2512 CpGs (Figure 1a). Next, we sorted the features by the level of significance and further iteratively generated RFR/LOOCV models, gradually increasing the number of analyzed variables from the single most significant up to the total 2512 CpGs. The average MAE and R^2^ values for all models were 0.865 and 0.803, respectively, and the best performance was achieved by the model based on 28 CpGs (MAE = 0.624, R^2^ = 0.898) (Figure 1b). Those CpGs were located in 22 genomic regions associated with genes which are involved in various biological processes, including the glucose metabolic process (*ADPGK*, *PDK2*), double-strand break repair (*SEM1*), anatomical structure morphogenesis (*ANKRD11*), and chromatin remodeling and organization (*DPF3*, *MEG3*), according to DAVID (The Database for Annotation, Visualization, and Integrated Discovery) (Table S2) [40].

Although we were unable to test the performance of the obtained microarray-based model on the independent test samples due to the limited size of the available dataset, this simulation still demonstrates that a limited set of microarray data might be suitable for determining the dependency of the predicted variable like cell passage number on the DNA methylation level of a limited set of CpGs. However, regarding minimized eAge, the analysis of a few dozen genomic regions is suboptimal and it is not evident as to how that amount might be further reduced to produce a compact and cost-effective method of analysis.

### 2.2. Using Targeted Bisulfite Sequencing to Build Minimized Epigenetic Clocks

To assess the conceptual applicability of targeted BS-seq methodology for minimized eAge clocks development, we generated 44 samples of cultured MSCs, including independently serially passaged umbilical cord MSCs (ucMSCs) and bone marrow MSCs (bmMSCs) from donors of different ages (Table S3). Continuous passaging was accompanied by characteristic traits of replicative senescence, including changes in the shape of the cell and nucleus, altered gene expression, and the increased activity of senescence-associated β-galactosidase (Figures S1 and S2).

To select target CpGs, we made a shortlist of ten candidate loci that were previously used for MSC passage number prediction (*ALOX12*, *DOK6*, *LTC4S*, *FPGT-TNNI3K*) or demonstrating age-dependent DNA methylation dynamics in various cell types (*ASPA*, *EDARADD*, *ELOVL2*, *FHL2*, *PDE4C*, *PENK*) (Table S4) [25,26,27,38,41,42,43]. After that, we filtered out the loci that did not meet the following criteria: (1) the genomic locus should contain CpGs whose DNA methylation level correlates with the duration of MSC culture in the generated BS-seq data; (2) the locus should contain stretches of at least five CpGs in the 150 bp region to allow for the calculation of DNA methylation heterogeneity scores and make the analysis affordable and effective by using 2 × 75 paired-end sequencing. Eventually, genomic regions in the vicinity of the *ALOX12*, *ELOVL2*, *FHL2*, and *PDE4C* genes met these criteria and, therefore, were used further (Figure S3).

**Figure 2:**
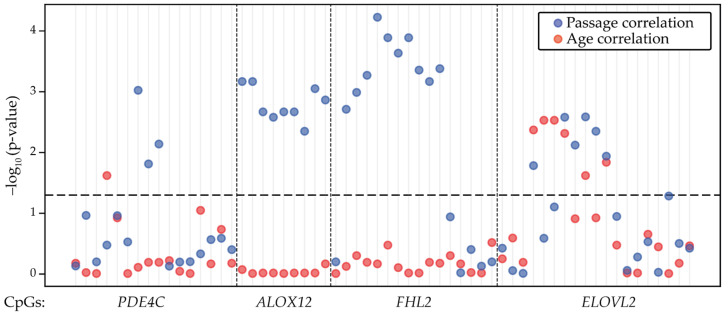
Significance of correlations between the average methylation level and cell passage (blue dots) or chronological age (red dots) for CpGs in the genomic regions selected for the BS-seq eAge model design. The dashed line indicates the 5% level of significance.

**Figure 3:**
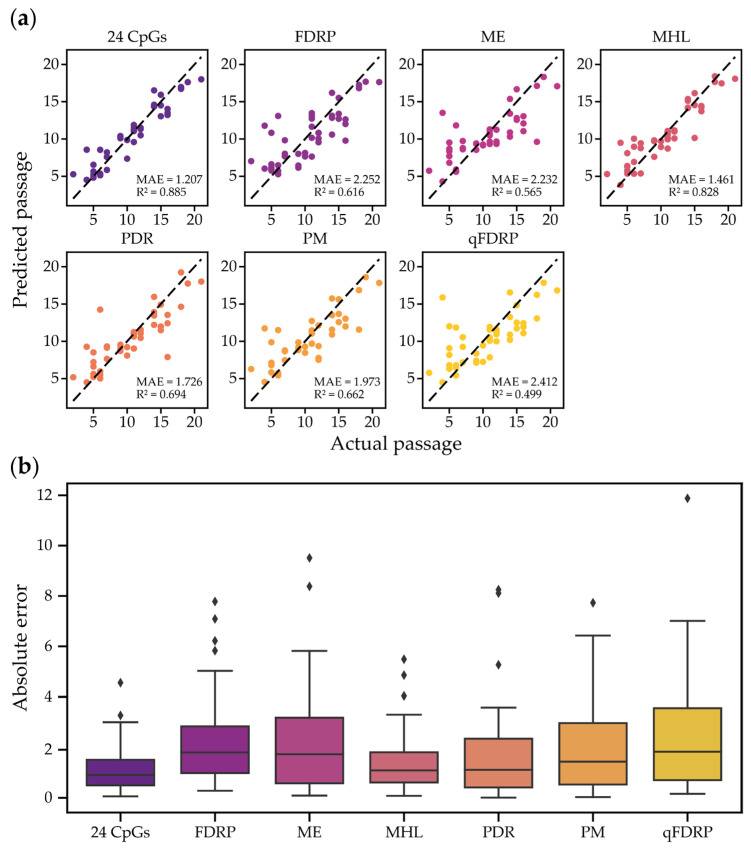
(**a**) The performance of BS-seq RFR models built using average DNA methylation values (24 CpGs model) and WSH scores (FDRP, ME, MHL, PDR, PM, and qFDRP). (**b**) BS-seq RFR models of absolute error distribution.

In total, the selected regions encompassed 60 CpGs, and subsequent targeted BS-seq analysis revealed that the DNA methylation level of 28 out of them showed a statistically significant correlation with the MSC cultural passage numbers (Pearson’s correlation, *p*-value adjusted < 0.05) (Figure 2, Table S5). As mentioned hereinabove, the DNA methylation of distinct CpGs in the vicinity of the *ELOVL2, FHL2*, and *PDE4C* genes correlates with the chronological age of different human tissues. To build a model solely capable of predicting cell passage, we aimed to exclude CpGs that might correlate with chronological age. Based on the BS-seq of bmMSCs obtained from donors of different ages, the DNA methylation levels of seven out of the sixty CpGs showed a dependence on chronological age: six of them were located in the *ELOVL2* gene region and a single one in the vicinity of *PDE4C* (Table S5). The DNA methylation of four CpGs in the *ELOVL2* locus significantly correlated with the donor’s chronological age, as well as with the cell culture passage number. Eventually, all of the CpGs that demonstrated chronological age-dependent DNA methylation dynamics were discarded, and the model built on the average DNA methylation values of the remaining 24 CpGs allowed us to predict the MSCs’ passage numbers with MAE = 1.207 and R^2^ = 0.885 (Figure 3a). The obtained performance was comparable with the benchmarked DNA methylation microarray-based predictive model and required the analysis of fewer genomic loci.

To further assess the reproducibility of cell passage prediction with the BS-seq and DNA methylation microarray data, we intersected the set of 24 CpG used as regressors in the BS-seq-based model with the Illumina Infinium HumanMethylation450 BeadChip array used to generate the datasets for the DNA methylation microarray-based model. Only 5 out of 24 CpGs were present in all the DNA methylation microarray datasets (*cg03760483*, *cg03404566*, *cg03762994*, *cg22454769*, *cg24079702*). Despite the low number of predictors, we still assessed the performance of the 5-CpGs models based on the DNA methylation values from the microarray and BS-seq data, demonstrating a comparable MAE of 1.330 passages, R^2^ = 0.552 and MAE of 1.568, R^2^ = 0.789, respectively (Figure S4). Although the number of predictors used is unlikely to be sufficient for reliable model performance, this assessment nevertheless qualitatively demonstrates an overall convergence of the results, regardless of the analysis method, and, to some extent, independently validates the selection of loci for MSC passage prediction.

### 2.3. Using DNA Methylation Heterogeneity Scores as a Predictor to Build BS-Seq-Based Minimized Epigenetic Clocks

Another advantage of the BS-seq method is that, alongside the analysis of the average DNA methylation of adjacent CpGs, it might be used to analyze the longitudinal DNA methylation patterns of every single read, thereby giving the opportunity to investigate DNA methylation heterogeneity. Previously, we showed that the analysis of DNA methylation heterogeneity using the set of specific WSH scores can be used to build eAge clocks on the datasets of reduced-representation bisulfite sequencing (RRBS) [31]. To assess the applicability of a similar approach for the design of a minimized targeted BS-seq eAge clock, we built the models to predict the MSC passage number using different WSH scores: MHL (Methylation Haplotype Load), PDR (Proportion of Discordant Reads), PM (Epipolymorphism) and ME (Methylation Entropy), FDRP (Fraction of Discordant Read Pairs), and qFDRP (quantitative Fraction of Discordant Read Pairs). Among these, MHL and PDR are specifically designed to measure the randomness of methylation patterns or erosion, and PM and ME measure the diversity of the methylation patterns within adjacent CpG quadruplets, while FDRP and qFDRP capture diversity at the level of individual CpG [28,29,44,45,46].

To assess the dynamics of DNA methylation heterogeneity in the four genomic regions used to build a BS-seq-based predictive model, we calculated the heterogeneity metrics for all of the encompassed CpGs. Next, we selected CpGs (FDRP, qFDRP) or CpG-blocks (MHL, PDR, PM, ME), demonstrating the strongest Spearman’s rank correlation with the cell passage numbers and used them as predictors to build RFR models (Tables S6–S11, Figure 3). The MHL-based model demonstrated the lowest MAE of 1.461 passages and the best coefficient of determination: R^2^ = 0.828. The models based on PDR and PM metrics performed weaker, with MAE = 1.726, R^2^ = 0.694, and MAE = 1.973, R^2^ = 0.66, respectively. The other metrics, such as FDRP (MAE = 2.252, R^2^ = 0.616), ME (MAE = 2.232, R^2^ = 0.565), and qFDPR (MAE = 2.412, R^2^ = 0.499) demonstrated the worst performance among the others. Therefore, in our case, the application of heterogeneity metric scores alone did not outperform the usage of the average DNA methylation levels of individual CpGs for building models to predict MSC passage. In general, WSH-based models tended to produce more outliers and exhibited lower R^2^ values (Figure 3a), consistent with our previous findings [31], where average DNA methylation-based models demonstrated better convergence compared to WSH-based approaches. Unlike average DNA methylation, which relies on individual CpG states, WSH metrics assess combinatorial DNA methylation patterns across overlapping sequencing reads, suggesting that the increased variability in WSH-based predictions may reflect sample-specific differences in methylation patterns that do not significantly influence mean methylation levels at individual CpGs. While further experiments are still required to elucidate the underlying causes of the higher experimental variation in WSH scores, we next sought to explore whether integrating heterogeneity metrics with mean methylation could enhance passage prediction accuracy.

### 2.4. Combining the Passage-Dependent Dynamics of WSH Scores and the DNA Methylation Level to Predict Cultural Passage

During in vivo and in vitro cellular aging, DNA methylation at specific CpG sites can change either stochastically or through gradual monotonic shifts [47,48]. Incorporating measurements that capture both types of methylation dynamics could improve the predictive power of regression models. Therefore, to test the BS-seq-based models, built on the simultaneous analysis of average DNA methylation levels and local heterogeneity patterns, we created hybrid datasets combining both parameters. Namely, we extracted the predictors used to build the above-mentioned BS-seq models that were based on the (1) average DNA methylation level of individual CpGs (24 CpGs), (2) MHL heterogeneity scores (43 CpGs), and (3) PDR heterogeneity scores (5 CpGs) (Tables S6, S9 and S10). Next, we combined those variables to create hybrid datasets, including the (1) average DNA methylation level and MHL scores, (2) average DNA methylation level and PDR scores, and (3) average DNA methylation level and MHL and PDR scores.

**Figure 4:**
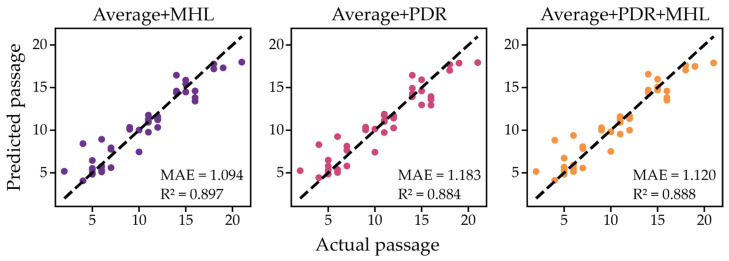
The performance of hybrid RFR models built on average DNA methylation values and combined with MHL and PDR heterogeneity scores as predictors.

Each combined dataset was used to build RFR-based models using LOOCV. The hybrid model combined data on the average DNA methylation, and MHL metrics performed the best, with MAE = 1.094 passages and R^2^ = 0.897 (Figure 4). Hybrid models combining average DNA methylation with PDR or MHL and PDR demonstrated a weaker performance, with MAE = 1.183, R^2^ = 0.884, and MAE = 1.120, R^2^ = 0.888, respectively. However, all of the combined models outperformed those that were solely based on DNA methylation heterogeneity metrics or average DNA methylation levels (Figure 3a and Figure 4). Therefore, we observed an improvement in the performance of the BS-seq-based cultural age prediction models when combining the analysis of average DNA methylation and heterogeneity metrics (Table 1).

**Figure 5:**
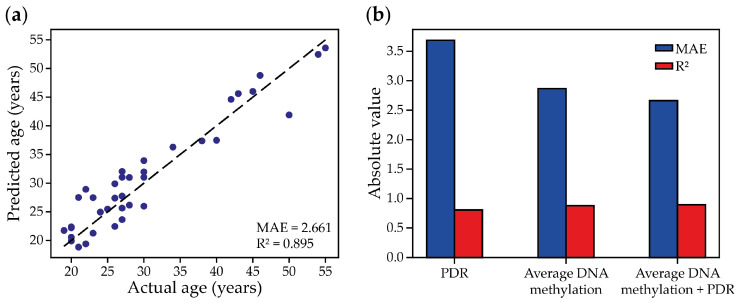
The performance of the chronological age prediction models (**a**) using average DNA methylation values and the PDR scores as predictors and (**b**) a comparison of the performance of models utilizing various DNA methylation-based predictors.

The small size of the dataset used to train the model could lead to overfitting and a reduced generalizability of the model. In this regard, for a more objective assessment, we decided to test the combined approach on the largest dataset of DNA methylation data to date, obtained by the high-throughput reduced-representation bisulfite sequencing (RRBS) of 182 whole-blood samples from donors of different ages [49]. In a previous study, we used it to build regression models estimating chronological age on the basis of average regional DNA methylation or WSH scores [31]. Among all the others, two regression models performed best in the prediction of chronological age. The first one analyzed the average DNA methylation data of 53 genomic 100 bp regions (MAE = 2.866 years, R^2^ = 0.877) (regional-eAge), and the second one analyzed the PDR scores of 48 CpGs located in only 6 short genomic regions (MAE = 3.686 years, R^2^ = 0.806) (PDR-eAge) [31]. To estimate the effect of the combined analysis of the average DNA methylation levels and patterns of heterogeneity on the precision of chronological eAge algorithms, we constructed a hybrid predictive model. To build it, the RRBS dataset was divided into a training (80%, 145 samples) and a test set (20%, 37 samples). For each RRBS sample, we calculated the regressors of the regional-eAge and PDR-eAge models, 101 in total, and used them to build a LASSO regression model. The efficiency of the hybrid eAge model outperformed the initial models, demonstrating a MAE of 2.661 years and R^2^ equal to 0.895 (Figure 5a). This suggests that the simultaneous analysis of DNA methylation levels and patterns of heterogeneity might be applied not only to targeted BS-seq but also to other high-throughput sequencing methods of DNA methylation analysis, like RRBS, to enhance the performance of eAge models.

## 3. Discussion

Epigenetic clock algorithms that analyze the dynamics of DNA methylation in various tissues and developmental contexts are considered a universal tool for assessing the integral effects of aging at the organismal and cellular levels [14]. Although widely used for the assessment of age or distinct biochemical or physiological parameters related to aging, the applicability of the most widely used eAge clock for the prediction of health- and age-related conditions, as well as the effect of cell rejuvenation, is currently debated [34,35]. Indeed, there are several conceptual and technical caveats that might greatly affect the accuracy and applicability of a single eAge model. Most eAge clocks are built on DNA methylation microarray data and use penalized regression methods, which can lead to batch effects [17]. These effects may be intrinsic (like age-related changes in cell composition) or technical (due to variations in DNA methylation analysis procedures) [34,35,50,51]. Altogether, this leads to a limited applicability of pre-trained eAge models to out-of-sample datasets [17]. It is also noteworthy that most published eAge models have been trained on the DNA methylation values of tissue samples composed of multiple cell types [52,53]. When there is a need to analyze the epigenetic age of a specific human cell type, or to study less investigated species, the development of custom epigenetic clock algorithms might be required [54]. When sufficient data are available, you can use a common framework of eAge design, although it will have the above-mentioned limitations. However, when the datasets for training and tests are scarce, a convenient and effective framework, as well as a cost-effective analysis pipeline, might be required to develop custom eAge models that are tailored to address specific questions.

Minimized eAge clocks that only analyze a few genomic regions might serve as the most cost-effective way to analyze epigenetic age [14,55]. Compared to full-sized models, minimized eAge models analyze the DNA methylation of only a few CpGs, highlighting the need for the thorough selection of marker CpGs and limiting the predictive power of the model, in general. Moreover, most of the minimized models use pyrosequencing or PCR variations to analyze the average DNA methylation level [14,26,27,56]. However, there is increasing evidence that DNA methylation changes are rather attributed to the epigenetic drift of a different nature and stochastically generated DNA methylation patterns or “DNA methylation noise” might serve as a more robust predictor of aging than differences in the average DNA methylation in single CpG [26,31,35]. In comparison to DNA methylation microarrays, pyrosequencing, or PCR, high-throughput sequencing might detect the average DNA methylation values and consecutive DNA methylation patterns of adjacent CpGs with a single-DNA-molecule resolution, thus providing the basis for calculating DNA methylation heterogeneity, which might be attributed to epigenetic drift, cell type composition, and epigenetic mosaicism [28,55,57].

Here, we tested the framework for a minimized eAge clock design based on targeted BS-seq using the in vitro replicative senescence of MSCs as an experimental model. The generated model was built on four genomic regions, ensuring a simple and affordable analysis. This approach enabled the analysis of the DNA methylation of multiple passage number-correlated CpGs, enhancing the performance of the model (MAE = 1.207 and R^2^ = 0.885). It is interesting to note that only around half of the CpGs located in the analyzed genomic regions correlated with the duration of the cell culture, thereby highlighting the previous notion that adjacent CpGs demonstrate uncoordinated DNA methylation dynamics during cell culture [26,55]. Eventually, the generated passage predictor performed comparably to the other known cultural eAge clocks. For example, the 4-CpG model, proposed in the work of Franzen et al. [26], demonstrates MAE = 2.4 passages and R^2^ = 0.81 on training data, while we managed to achieve MAE = 1.094 and R^2^ = 0.897. However, it is worth mentioning that the 4-CpG model was built to predict the passage of MSCs, HUVECs, and fibroblasts, and it was trained on pyrosequencing data, thereby making a direct comparison difficult [26]. Moreover, by combining DNA methylation heterogeneity metrics with average DNA methylation dynamics, we developed a model that outperformed the conventional approach that solely relies on methylation values. Moreover, we validated that the combined analysis of DNA methylation heterogeneity and average methylation might be beneficial for chronological eAge clock design. This approach was applied to an RRBS dataset of 182 whole-blood samples from donors of different ages, generating a model with MAE = 2.661 years, which is within the lower end of the MAE range for analogous published DNA methylation microarray-based models [14,49].

Although further experiments, using various cell types or assessing the detection of a factor that might influence aging or senescence, are required to directly prove the effectiveness of the framework, we have provided a mechanistic conceptualization of how characteristic, more complex changes in DNA methylation patterns during replicative senescence could potentially be used to assess it. However, it is important to point out several limitations of the eAge predictive models. An intrinsic limitation is that eAge regression models are tailored to predict the dependent variables included in the training set, such as chronological age, morbidity risk, and physiological data, in case of studying organism aging [14]. For an in vitro replicative senescence study, the prediction of the passage number, reflecting the time in culture, might be considered the simplest parameter, which is inevitably positively correlated with the replicative senescence marks. To the best of our knowledge, current senescence or mitotic eAge models are unable to detect “immediate” senescence, such as stress or oncogene-induced senescence types [58,59,60]. This limitation might be explained by the prevailing hypothesis considering epigenetic drift to be the major cause of eAge methylation dynamics, and that is why its manifestation might be directly linked to the number of cell divisions or time span [26]. However, it is tempting to assume that including functional dependent variables in model training, like cell morphology or quantitative information of characteristic gene expression, might allow for the prediction of senescence-related cell features directly. Given the evolution of human eAge clocks, starting from chronological age prediction to advanced algorithms capturing aging outcomes, the development of multi-parameter-based minimized eAge models predicting functional cell state sounds like a perspective direction. However, this will require the generation of datasets complemented with the various kinds of functional cell properties related to replicative senescence and the proper design, including the selection of appropriate genomic regions demonstrating the most informative DNA methylation dynamics. The development of such predictive algorithms may be beneficial for aging and cancer risk prediction in a manner similar to epigenetic mitotic clocks, considered now a prospective tool for precancerous diagnostics [48]. Finally, our data suggest that distinct CpGs within the same genomic region may exhibit divergent associations with the cell passage number and donor age. While the biological mechanisms underlying this observation remain to be explored, it might be proposed that these patterns could form the basis for a novel tool capable of concurrently evaluating both the chronological (donor) and replicative (culture) age of cells.

## 4. Materials and Methods

### 4.1. Source Data

The methylation data of 48 samples of HumanMethylation450 BeadChip [37,38] were downloaded from the GEO database. A list of the samples used is provided in Table S1. We also used data from the bisulfite sequencing of 182 whole-blood samples downloaded from the ENA database via BioProject identifier PRJNA531784 [49].

### 4.2. Donor MSCs

All of the human cell samples were taken with the voluntary informed consent of the donors. This study was approved by the local ethics committee of the Federal Center for Medical Sciences of the Federal Medical and Biological Agency of Russia (protocol No. 7-5-22 dated 6 September 2022). Umbilical cord MSCs (*N* = 2) were isolated from Wharton’s jelly from a healthy mother at 38–40 weeks of gestation. A 3–4 cm fragment of the umbilical cord from the placental side was cut with sterile scissors, washed with DPBS solution (Gibco, Waltham, MA, USA), minced, and then incubated with 1% collagenase type I solution (Gibco, Waltham, MA, USA) for 8 h at 37 °C. The cell suspension was diluted tenfold in DPBS, centrifuged (400× *g*, 4 min), and the obtained cell preparations were further expanded in αMEM-Glutamax growth medium without nucleosides (Gibco, Waltham, MA, USA), 100 U/mL penicillin, 0.1 mg/mL streptomycin (Gibco, Waltham, MA, USA) and 4% human platelet lysate (StemCell Technology, Vancouver, BC, Canada) in a multi-gas incubator at 37 °C, 5% CO_2_, and 5% O_2_ in culture flasks with a growth surface area of 175 cm^2^. Every three days, the cell medium was changed to a new one. When 90% of the monolayer density was reached, the cells were subcultured by trypsinization.

Bone marrow MSCs (*N* = 6) were isolated from the donor-derived bone marrow mononuclear cell fraction obtained by gradient centrifugation (20 min, 400× *g*) in Ficoll solution (PanEco, Leninskiye Gorki, Russia) and were then cultured in DMEM/F-12 cell medium (Gibco, Waltham, MA, USA) with 10% FBS (Gibco, Waltham, MA, USA), 100 U/mL penicillin, and 100 μg/mL streptomycin (Gibco, Waltham, MA, USA) at 37 °C and 5% CO_2_. The medium was changed every 3 days. When 90% confluency of the monolayer was reached, the cells were subcultured by trypsinization.

All of the cell preparations were tested for compliance with the required MSC criteria [61] using several methods: characterization of morphology, trilineage differentiation with hMSC Differentiation Kits (Gibco, Waltham, MA, USA, #A1007001, #A1007101, #A1007201), and flow cytometry with anti-CD29/CD44/CD73/CD90/CD105/CD34/CD45 antibodies (Miltenyi Biotec, Bergisch Gladbach, Germany, #130-118-121, #130-113-342, #130-111-908, #130-114-860, #130-112-169, #130-120-515, #130-110-631, all labeled with FITC/PE).

When passaging, the MSCs were cultured in DMEM/F-12 cell medium (Gibco, Waltham, MA, USA) with 15% FBS (Capricorn, Edinburgh, UK, FBS-11A), 100 U/mL penicillin, and 100 μg/mL streptomycin (Gibco, Waltham, MA, USA) at 37 °C and 5% CO_2_. The cells were cultured up to 90% confluency and passaged in a ratio of 1:4. The increase in SA-b-gal expression was measured as described previously [62]. Dynamics of the gene expression levels of *CDKN2A* (*P16INK4A*), *CDKN1A* (*P21*), *LMNB1*, and *HMGB2* were tested by reverse transcription real-time PCR. Briefly, the total RNA was extracted using RiZol reagent (diaGene, Moscow, Russia, #3789.0250) and used for cDNA synthesis with a reverse transcription kit (Biolabmix, Novosibirsk, Russia, #R03-10). Real-time PCR was performed with BioMaster UDG HS-qPCR SYBR Blue (Biolabmix, Novosibirsk, Russia, #MHC031-2040) and the primers are listed in Table S12.

### 4.3. Targeted Bisulfite Sequencing

Genomic DNA was isolated using the QIAamp DNA Mini Kit (Qiagen, Venlo, The Netherlands, #51306) and bisulfite was converted with the EZ DNA Methylation-Gold Kit (Zymo Research, Irvine, CA, USA, #D5006). Next, the DNA regions of interest were amplified using GoTaq DNA polymerase (Promega, Madison, WI, USA, #M300A) using the primers listed in Table S13, purified with KAPA HyperBeads (KAPA Biosciences, Wilmington, MA, USA, #08963851001), and quantified with Qubit Fluorometer (ThermoFischerScientific, Waltham, MA, USA). Then, the PCR products of the same template origin were pooled equimolarly. To prepare barcoded NGS libraries, pools were subsequently indexed by PCR with KAPA HiFiReady Mix (KAPA Biosystem, Wilmington, MA, USA, #07958927001) and Nextera CD DNA Indexes (Illumina, San Diego, CA, USA, #20018708). Indexed libraries were analyzed via 2 × 75 bp paired-end sequencing on Illumina MiSeq (Illumina); more than 20,000 reads (median~110,000 reads) were generated per sample, ensuring a high coverage of every locus analyzed.

### 4.4. Microarray Data Analysis

All HumanMethylation450 BeadChip datasets were merged into a single unified dataset. To reduce the dimensionality of the dataset and improve model performance, we filtered the CpG sites using the Pearson correlation coefficient (*p*-value adj < 0.05). To determine the optimal number of CpG sites at which the model would perform best, we applied the following approach: a Random Forest Regression (RFR) was built using the Scikit-learn v1.4.0 Python package [63]. Due to the limited number of samples and the necessity to reduce the risk of overfitting, the Leave-One-Out Cross-Validation (LOOCV) approach was employed. To identify the CpG sites with the highest significance in the model, we calculated the cumulative significance of each CpG site across all cross-validation iterations, after which we removed the features with zero importance. Next, all of the CpG sites were sorted in ascending order of their importance. Starting with the single most significant feature and gradually increasing their number up to the maximum, we iteratively built RFR models with LOOCV to evaluate their performance.

### 4.5. Sequencing Reads Processing and Heterogeneity Calculation

Read quality control and filtration was performed using TrimGalore v0.6.10 [64]. Filtered reads were mapped to the human reference genome GRCh38 using Bismark v0.24.2 with Bowtie 2 v2.5.2 [65,66]. Position-sorted BAM files were then used for heterogeneity metric calculation (FDRP, ME, MHL, PDR, PM, qFDRP) by Metheor v0.1.8 [67].

### 4.6. Selection of Genomic Features for the Minimized Predictive Model

The procedure for selecting four loci and screening for the CpGs correlated with passages was conducted as follows: At the first stage, we sequenced 10 loci (in the vicinity of *ALOX12*, *DOK6*, *LTC4S*, *FPGT-TNNI3K*, *ASPA*, *EDARADD*, *ELOVL2*, *FHL2*, *PDE4C*, and *PENK*) in the bmMSC and ucMSC preparations (*N* = 9) (Table S3). Next, we examined Pearson’s correlations between average methylation, donor age, and culture passage, as described in Section 2.2 of the Results (Figure S2). We then selected the genomic regions in the vicinity of the *ALOX12*, *ELOVL2*, *FHL2*, and *PDE4C* genes and sequenced additional MSC samples. Finally, by analyzing the correlation between the average DNA methylation and age indicators in fifteen MSC preparations (Table S3, “Used for CpG selection” column), we selected 24 CpG positions that significantly correlated with passage, but not with donor age, which were used as predictors for the RFR model (Figure 2).

### 4.7. Building Predictive Models with RFR

A complete targeted BS-seq dataset containing the average methylation values at the selected CpG sites for MSC preparations was used as the initial data (Table S3). To build predictive models, RFR with the LOOCV algorithm was used. In the framework of the LOOCV method, the model was recursively trained on all samples except one, which was used for testing. This process was repeated for each sample in the dataset. The quality of prediction was assessed with MAE and R^2^ metrics.

### 4.8. Implementation of the Hybrid Model of Cultural Age

To implement a hybrid model of cultural age clocks, we combined the data in three different ways: highly correlated with cultural age CpG sites and (1) MHL regions, (2) PDR regions, and (3) combined MHL and PDR regions. To build the model, we used the same approach: the RFR model with LOOCV.

To validate the approach, we used the data that we had previously obtained based on a published dataset of reduced-representation bisulfite sequencing of 182 blood samples [49]. Fifty-three significant CpG regions, each of which was 100 bp in size, were combined with the best 48 CpGs for PDR metrics correlating with the chronological age of the blood donors. The model construction was implemented in a similar way to the previous study [31]. In short, the dataset was divided into training (80%) and test (20%) samples. The alpha hyperparameter in LASSO regression was selected using tenfold cross-validation on the training set. The best hyperparameter alpha was then used to train the model, which was subsequently evaluated on the test data.

## Figures and Tables

**Table 1:** Comparative performance of the BS-seq RFR models built in the present study.

Model	R^2^	MAE
Average methylation in 24 CpG	1.207	0.885
FDRP	2.252	0.616
ME	2.232	0.565
MHL	1.461	0.828
PDR	1.726	0.694
PM	1.973	0.662
qFDRP	2.412	0.499
Average + MHL	1.094	0.897
Average + PDR	1.183	0.884
Average + MHL + PDR	1.119	0.888

## Data Availability

The datasets generated during the current study are available from the corresponding author on reasonable request.

## Supplementary Materials

The following supporting information can be downloaded at https://www.mdpi.com/article/10.3390/ijms26115051/s1.
