# Supplementary material for: A Novel Framework for the Design of Minimized Epigenetic Clocks Using the Analysis of DNA Methylation Heterogeneity
Source: Int J Mol Sci. 2025 May 23;26(11):5051. doi: 10.3390/ijms26115051 (PMC12155483; doi:10.3390/ijms26115051)
Supplement: Supplementary file 1 [file ijms-26-05051-s001.zip › Supplementary Figures.pdf]

SUPPLEMENTARY FIGURES

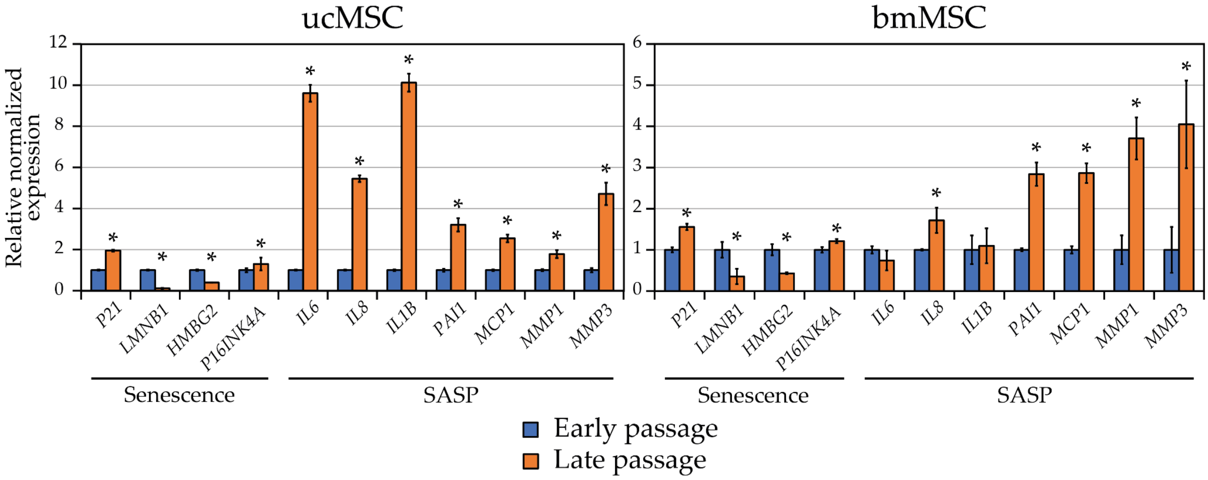

Figure S1. RT-qPCR measurements of relative normalized expression of senescence and SASP (Senescence Associated Secretory Phenotype) marker genes in cultured ucMSCs/bmMSCs used for targeted BS-seq. Cells in early passages were used as a control. As expected, during continuous cell culture, the cells experience a decrease in expression of mRNA encoding nuclear proteins HMGB2 and LMNB1, an increase in the activity of proliferation arrest markers p21 and p16ink4a, and an in-crease in the expression of genes involved in SASP. * - P-value < 0.05 (Student's t-test).

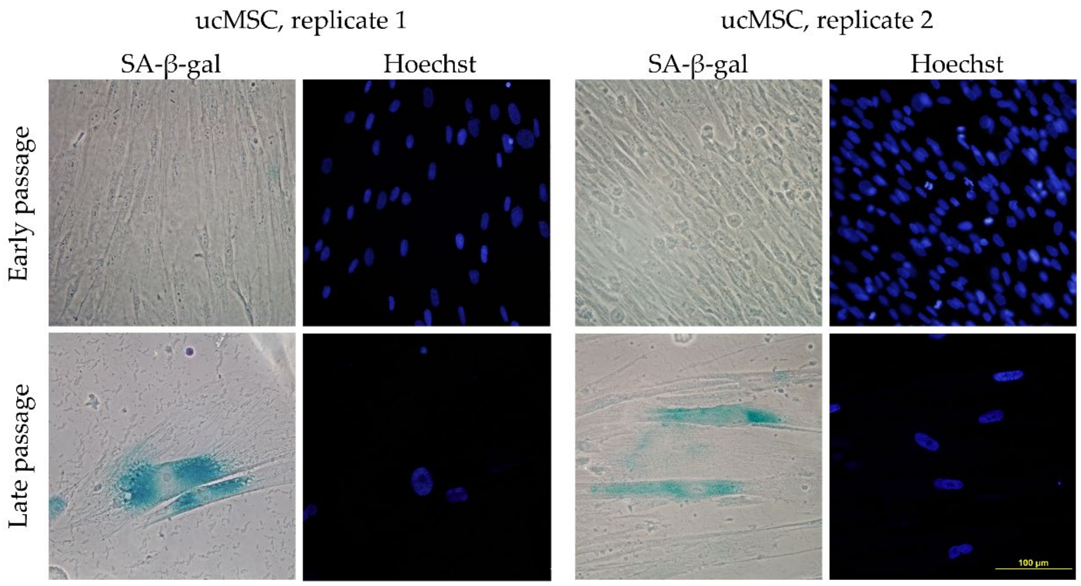

Figure S2. SA-β-gal and Hoechst staining of cultured ucMSCs, used for targeted BS-seq. Cells on early and late passages are presented in 2 biological replicates. As expected, during long-term culture MSCs undergo an increase in size and nuclear area and acquire SA-β-gal-positive staining. Scale bar - 100 µm.

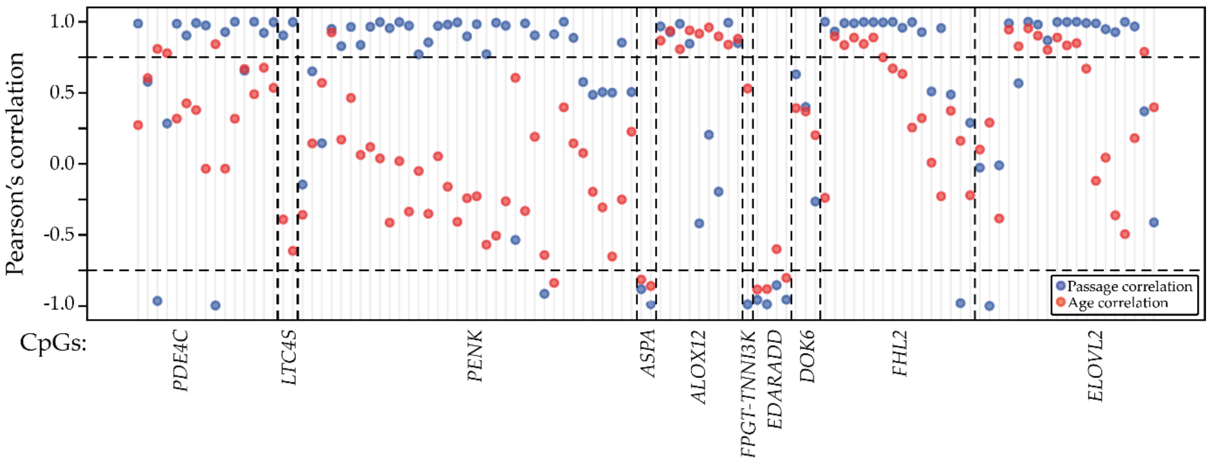

Figure S3. Correlation of average CpG methylation with donor's age and culture passage at 10 selected loci according to targeted BS-seq of cultured MSCs samples.

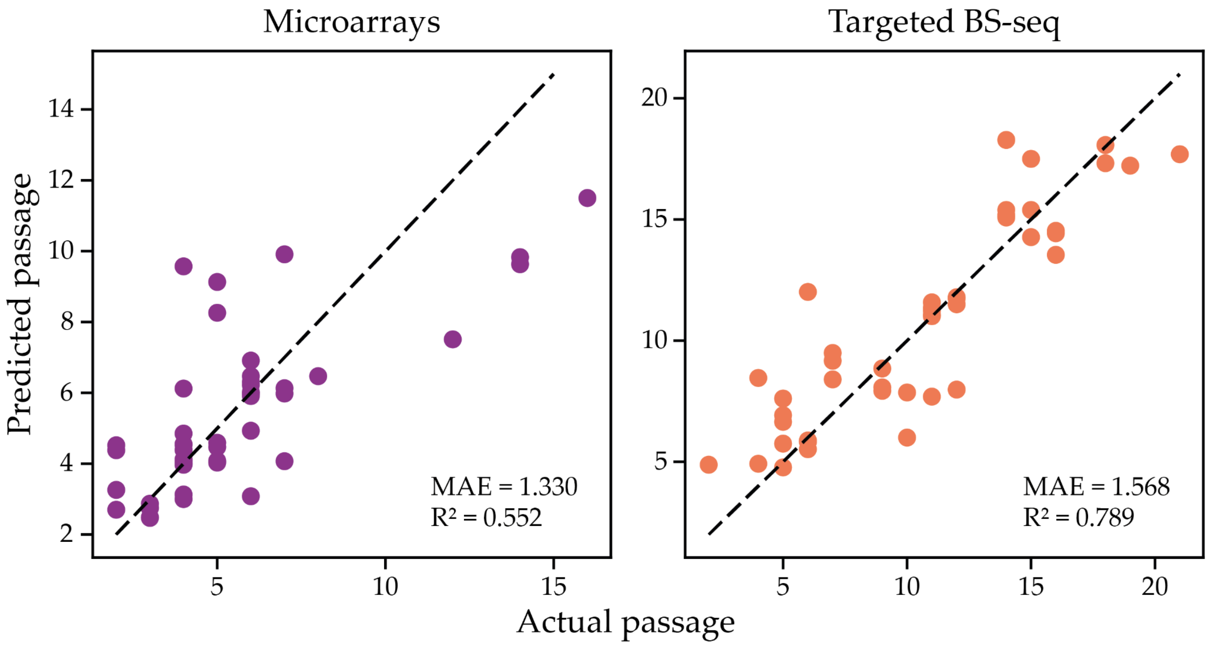

Figure S4. The performance of 5 CpGs RFR models based on DNA methylation values of Human Methylation 450 BeadChip dataset and the DNA methylation values generated by targeted BS-seq.
